# Individual and country-level factors associated with self-reported and accelerometer-based physical activity in old age: a cross-national analysis of European countries

**DOI:** 10.1007/s10433-022-00737-8

**Published:** 2022-10-21

**Authors:** Tiia Kekäläinen, Martina Luchetti, Damaris Aschwanden, Angelina R. Sutin, Antonio Terracciano

**Affiliations:** 1 Gerontology Research Center, Faculty of Sport and Health Sciences, University of Jyväskylä, Jyväskylä, Finland; 2 Department of Behavioral Sciences, College of Medicine, Florida State University, Tallahassee, FL USA; 3 Department of Geriatrics, College of Medicine, Florida State University, Tallahassee, FL USA

**Keywords:** Physical activity, Personality, Quality of life

## Abstract

This study aimed to investigate associations between individual-level (personality traits, quality of life) and country-level (gross domestic product per capita, number of policies and action plans for physical activity) factors with self-reported and accelerometer-based physical activity and cross-level interactions among European countries. Based on the Survey of Health, Ageing and Retirement in Europe (SHARE) from 2019–2020, self-reported physical activity (*N* = 46,617 from 27 countries) and accelerometer-based average acceleration and intensity gradient (*N* = 855 from 10 countries) were analyzed. Mixed-model regressions with two levels (individuals nested within countries) were used for analyses. Between countries differences accounted for relatively small portions of the variability in self-reported physical activity (intraclass correlation, ICC = 7.5%), average acceleration (ICC = 3.5%), and intensity gradient (ICC = 1.9%). There were more associations between individual- and country-level factors and self-reported physical activity than with accelerometer-based physical activity. The association between individual-level variables and accelerometer-based physical activity did not differ between countries. Cross-level interactions suggested that associations between some personality traits and self-reported physical activity were stronger in countries with lower GDP. Both individual- and country-level factors are related to participation in more intensive physical activities. Adults with less resilient personality traits living in countries with lower resources are at the highest risk for physical inactivity.

**Supplementary Information:**

The online version contains supplementary material available at 10.1007/s10433-022-00737-8.

## Introduction

Engaging in physical activity is one of the most promising ways to prevent non-communicable diseases and age-related loss in functional capacity (Lee et al. 2012; Bauman et al. 2016; Moreno-Agostino et al. 2020). Still, the proportion of individuals with insufficient physical activity is high and increases with age (Dumith et al. 2011; Guthold et al. 2018; Lübs et al. 2018; Du et al. 2019). Physical activity is a complex behavior that is an interaction between individual factors and the environment (Sallis et al. 2006). Determinants of physical activity occur at multiple levels, such as intrapersonal, interpersonal, environmental, regional, national, and global levels (Bauman et al. 2012), and thus a multi-level viewpoint is needed to better understand physical activity behavior (Sallis et al. 2006).

Previous studies on multi-level determinants of physical activity have mainly focused on associations between individual-level socio-cognitive (e.g., self-efficacy, social support) or demographic factors and neighborhood environmental level (e.g., perceived walkability) (Carlson et al. 2012; Ding et al. 2012; Jürgens and Schüz 2021). Even though results vary depending on the variables of interest, they suggest cross-level interactions. The positive association between high self-efficacy and leisure walking, for example, seems to be stronger when facilities for walking are poorer (Carlson et al. 2012; Ding et al. 2012). Similarly, lower household income is a risk factor for physical inactivity only when the environment is less favorable (Jürgens and Schüz 2021). Some evidence, however, suggests the opposite interaction between socio-cognitive predictors and regional gross domestic product per capita (GDP), with stronger associations between socio-cognitive predictors and physical activity in regions with higher GDP (Schüz et al. 2012).

Less attention has been given to the interaction between the environment and more stable and pervasive individual-level factors, such as personality or quality of life which are known correlates of physical activity (Rejeski and Mihalko 2001; Wilson and Dishman 2015; Sutin et al. 2016). Personality traits are relatively stable over the lifespan (Roberts and Mroczek 2008) and may explain why some individuals are physically active and others are not within the same environment. Of the Five Factor Model personality traits, the most consistent associations with physical activity have been found for lower neuroticism and higher extraversion and conscientiousness, and to a lesser extent for higher openness (Wilson and Dishman 2015; Sutin et al. 2016; Wilson and Rhodes 2021). The interaction between personality traits and the environment has not been previously studied in the context of physical activity, but a recent study showed that the association between personality traits and cognitive function is stronger in countries with lower GDP, which suggests personality may work as a psychological resource when living in economically less advantageous nations (Luchetti et al. 2021). Quality of life refers to an individual´s overall perception of their position in life (Skevington et al. 2004) and is closely related to many resources in life, such as better health and socioeconomic situation (Conde-Sala et al. 2017). The association between quality of life and physical activity is often investigated from the viewpoint of how physical activity improves quality of life (Marquez et al. 2020), but quality of life may also be seen as a determinant of physical activity (Gimeno-Santos et al. 2014; Rhodes et al. 2017).

Of different environmental levels, the macro-level environment, including country-level indicators, is less studied in relation to physical activity. The macro-level environment interacts with other levels and may affect physical activity behavior also through all other levels (Cameron et al. 2013). Of macro-level variables, the level of economic development captured by GDP has shown the strongest association with physical activity: the percentage of the population that is physically active in their leisure time is higher in countries with higher GDP (Van Tuyckom 2011; Cameron et al. 2013). However, this difference is only apparent for leisure time physical activity; the association with overall physical activity has been either nonsignificant (Cameron et al. 2013; Laverty et al. 2018) or even negative (Bosdriesz et al. 2012). Higher GDP may reflect better infrastructure for physical activity, such as better biking paths and fitness centers, but also more sedentary occupations (Schüz et al. 2012; Cameron et al. 2013). In addition to GDP, a country´s overall environment for physical activity is dependent on policies for physical activity. Policies for physical activity may regulate laws, affect design of environments and possibilities for active transportation, provide funding for physical activity promotion, or promote various programs and national campaigns, (e.g., Moving Toward Obesity Solutions: Workshop Summary 2015). Policies reach a large proportion of the population and indicate the overall culture for physical activity (Moving Toward Obesity Solutions: Workshop Summary 2015).

Most previous studies on individual-level and especially macro-level are based on self-reported physical activity. The moderate-to-low correlations between self-reported and device-based physical activity (Kowalski et al. 2012) suggest that they capture partly different aspects of physical activity. Typically, accelerometers and other devices record both planned and incidental physical activity (including both work and leisure time) whereas self-reports capture mostly structured physical activities (Strath et al. 2013; Schrack et al. 2016). Previous studies have shown stronger associations between personality traits and self-reported physical activity than accelerometer-based physical activity (Wilson et al. 2015; Kekäläinen et al. 2020a, b) whereas health-related quality of life had a stronger association with accelerometer-based physical activity in one study (Anokye et al. 2012). To our knowledge, macro-level indicators are hardly studied with device-based physical activity. Because individuals in different environments could have different standards to be physically active and respond to physical activity questionnaires in different ways (Kapteyn et al. 2018), device-based measures of physical activity are critical for country-level comparisons to have a comprehensive understanding of physical activity in different environmental contexts.

The purpose of this study was to investigate 1) the association between individual-level (personality traits and quality of life) and country-level (GDP and the number of policies and action plans for physical activity) factors and self-reported and accelerometer-based physical activity, and 2) whether country-level factors moderate the association between individual-level factors and physical activity. Based on previous literature, we hypothesized that all individual- and country-level factors are associated with self-reported physical activity but the association with accelerometer-based physical activity may be weaker. We also hypothesized the association between individual-level factors and physical activity will be stronger in countries with lower GDP.

## Methods

### Study design and participants

The data from the Survey of Health, Ageing and Retirement in Europe (SHARE) and its accelerometer sub-study were used. SHARE is a cross-national multidisciplinary database from samples of community-based adults aged 50 or older (Börsch-Supan et al. 2013). The first data collection was in 2004 and new waves have been collected every two years. All respondents who were interviewed in any previous wave are part of the longitudinal sample. In the present study, we utilized accelerometer and questionnaire data from wave 8 (collected 2019/2020, Börsch-Supan 2022a) and questionnaire data on personality traits from wave 7 (collected 2017/2018, Börsch-Supan 2022b). In wave 8, 46,733 participants from 27 countries participated in the study: Austria, Belgium, Bulgaria, Croatia, Cyprus, Czech Republic, Denmark, Estonia, Finland, France, Germany, Greece, Hungary, Israel, Italy, Latvia, Lithuania, Luxembourg, Malta, Netherlands, Poland, Romania, Slovakia, Slovenia, Spain, Sweden, and Switzerland. The data collection started in October 2019 and was suspended in March 2020 because of the COVID-19 outbreak (Bergmann and Börsch-Supan 2021). On average, about 70% of all expected interviews for the longitudinal sample were completed at this point, but the state of fieldwork varied across countries (Scherpenzeel et al. 2020). A total of 46 617 participants from 27 countries had information on self-reported physical activity from wave 8.

For the accelerometer sub-study, data were collected from a subsample in 10 SHARE countries including two northern (Denmark, Sweden), two southern (Italy, Spain), three eastern (Czech Republic, Poland, Slovenia), and three central (Belgium, France, Germany) European countries. The sampling strategy targeted a net sample of 200 participants from each country. The gross sample was a stratified sample selected from each country’s longitudinal sample stratified by age group and self-reported physical activity levels in previous waves (Bergmann and Börsch-Supan 2021). The willingness to participate in the accelerometer study was asked during the main interview and if a participant was willing to do so, an accelerometer was mailed to them with instructions. The consent rate was on average 54.4%, and ranged from 33.7% (Czech Republic) to 70.2% (Poland) (Bergmann and Börsch-Supan 2021). The data collection was terminated early because of the COVID-19 outbreak. The final accelerometer sample included 856 participants.

### Measures

Self-reported physical activity. Self-reported physical activity was asked with questions about the frequency of vigorous physical activity (such as sports, heavy housework, or a job that involves physical labor) and moderate physical activity (such as gardening, cleaning the car, or doing a walk). The response options were 1 = More than once a week, 2 = Once a week, 3 = One to three times a month, and 4 = Hardly ever, or never. The response scale was reversed so that higher values indicated more physical activity and the mean for two questions was calculated (range 1–4) (cf. Cheval et al. 2021).

Accelerometer-based physical activity*.* Participants were asked to wear a tri-axial accelerometer (Axivity AX3, Axivity Ltd, Newcastle upon Tyne, United Kingdom) on their upper thigh for eight consecutive days all day and night (Bergmann and Börsch-Supan 2021). The accelerometers were set to a sampling frequency of 50 Hz (with a range of ± 8 g). Raw accelerometer data were processed in SHARE central with GGIR (Migueles et al. 2017), an open-source package for the statistical computing software R (R Core Team, 2020). Raw accelerometer data were aggregated to 5-s epochs and auto-calibrated (van Hees et al. 2013, 2014). Non-wear time was detected by bouts of 15 min. Data from days with at least 16 h of wear-time were included in the analyses.

Outcomes were average acceleration over a measurement period and intensity gradient (SHARE Release guide 8.0.0.^2022^). The average acceleration was calculated as the average vector magnitude per total measurement period as Euclidian norm minus one (ENMO) with negative values set to zero (van Hees et al. 2013) and presented in milligravity units. It indicates overall physical activity level over the measurement period and is comparable across studies and populations. The intensity gradient was based on a gradient from the log–log regression line between time and intensity. The more negative the gradient, the longer the time spent in sedentary activities and the shorter the time spent in more intensive activities. Thus, a more negative gradient indicates a worse intensity profile (Rowlands et al. 2018, 2022).

Personality traits. The 10-item Big Five Inventory (BFI-10) was used to measure neuroticism, extraversion, openness, agreeableness, and conscientiousness (Rammstedt and John 2007). The survey was developed to assess the five personality traits for contexts in which time is limited (e.g., large population studies). The BFI-10 has demonstrated good reliability and validity across different samples (test–retest correlations ≥ 0.65 across scales; Rammstedt & John, 2007). The response scale was from 1 = disagree strongly to 5 = agree strongly, and the score was reversed for five of the items. Each trait is assessed by two items (e.g., “I see myself as someone who is outgoing, sociable” for extraversion) and the mean for each trait was calculated in the direction of the trait label (Mehrbrodt et al. 2019).

Quality of life. The CASP-12 is a shortened version of the original CASP-19 Scale (Hyde et al. 2003). It includes four dimensions (Control, Autonomy, Self-realization, and Pleasure), and three questions assessed for each dimension (e.g., “How often do you think that you can do the things that you want to do?” for autonomy). The response options were 1 = Often, 2 = Sometimes, 3 = Rarely, and 4 = Never. The response scale was reversed for positive items and a sum score for 12 items was calculated (range 12–48) (Mehrbrodt et al. 2019). A higher score indicates a higher quality of life.

Country-level indicators. Gross domestic product (GDP) per capita and the number of national policies or action plans for the promotion of physical activity for health were used as country-level indicators. GDP from the year 2020 was collected from the World Bank (https://data.worldbank.org/indicator/NY.GDP.PCAP.CD). To facilitate interpretation of model estimates, GDP/10,000 was used in the analyses. The number of national policies and action plans was collected from the World Health Organization latest physical activity country factsheets (WHO 2021). For Switzerland, only information from previous factsheet wave (2018) was available, and there was no information available for Israel.

Covariates. The background variables included age (years), sex (0 = male, 1 = female), education, body mass index (BMI), chronic diseases, and the pandemic restrictions. The 1997 International Standard Classification of Education with seven categories (from 0 = none to 6 = second stage of tertiary education) was used in SHARE to harmonize education categories across European countries (UNESCO Institute for Statistics 2006). BMI was calculated from self-reported weight and height (kg/m^2^). The number of chronic diseases was calculated from a list of 21 common diseases or conditions (e.g., high blood pressure, high cholesterol, diabetes, cancer, osteoarthritis). Participants reported conditions that a doctor had diagnosed them with, and they were currently either treated for or bothered by the condition. As some (*n* = 73, 8.5% of the total sample) of the accelerometer measurements were taken after the onset of the COVID-19 pandemic, the country-specific situation during the measurement period was captured (Hale et al. 2021, 2022). A binary variable (0 = no measures, 1 = recommended or required not leaving the house) was used in the sensitivity analyses.

### Statistical analysis

All analyses were conducted using SPSS 26.0 software (IBM Corp, Armonk, New York, United States) and figures were created in RStudio 2022.02.0 (Boston, MA). The Netherlands was excluded from analyses with personality traits because personality was available only from 3.6% (*n* = 69) of the sample. Romania was excluded from the analysis on quality of life and Israel was excluded from analyses on the number of policies as this information was unavailable. Due to missing values in predictors and covariates, the analytic sample varied between 39,750–44,858 for self-reported physical activity and between 821–851 for accelerometer-based physical activity. Missing values were expected to be missing at random.

Linear multilevel modeling was used for the analyses because the data included individuals (level 1) nested within countries (level 2). The same set of analyses was performed for each outcome: average acceleration, intensity gradient, and self-reported physical activity. Because the interest was in the associations between each individual predictor and outcomes, and to avoid suppression effects, all factors predicting physical activity outcomes were analyzed in separate models.

First, the intraclass correlation coefficient (ICC) that assessed the proportion of variance in physical activity between countries was examined in a null model with no other variables. Likelihood ratio tests that compared a null multilevel model (including a random intercept) and a null single-level model were used to test the significance of country effects.

Second, the associations between predictors and physical activity outcomes were tested in models that included the predictor variable and demographic covariates (centered age, gender, and education) (Model 1). To test whether these associations varied between countries, both a random-intercept-only model and a random-intercept-random-slope model were estimated. In the latter, a random slope allowed the association between an individual-level predictor and physical activity outcome to vary between countries and an unstructured covariance matrix was used. Likelihood ratio test model comparisons were used to test whether the random slope improved the model fit. If the likelihood ratio test (*p* > 0.05) indicated that the random slope did not improve model fit or model convergence problems indicated the lack of random effects in the data, the random-intercept-only model was used as a final model (Meteyard and Davies 2020). If the likelihood ratio test was statistically significant (*p* < 0.05), the random-intercept-random-slope model was chosen as the final model. Additional models that included health-related covariates were estimated to test whether the results were similar after adjusting for chronic diseases and BMI (Model 2). A model including all personality traits and covariates (centered age, gender, education, chronic diseases, and BMI) simultaneously (Model 3) and a model including all predictors (personality traits, quality of life, GDP, the number of policies) and covariates simultaneously (Model 4) were also estimated for each outcome. These models evaluate whether each trait association with the outcomes is independent of the other traits (Model 3) and whether the associations hold when accounting for quality of life and country-level factors (Model 4).

Third, if the previous step suggested that the associations between individual-level factors and a physical activity outcome varied between countries, the moderator effects of country-level factors were investigated by adding a country-level factor and an interaction term between an individual-level factor and a country-level factor (e.g., neuroticism*GDP) to the model. Covariates were added in two steps as described above (Model 1 and 2) and an additional model including all interaction terms between personality traits and a country-level factor simultaneously was estimated (Model 3).

Two sets of sensitivity analyses were done. First, the analyses for self-reported physical activity were repeated restricting the sample to those who also had accelerometer data (*n* = 856) to examine whether the results in the large sample can be replicated in the accelerometer sample. Second, the analyses for accelerometer-based outcomes were repeated after the exclusion of participants (*n* = 73) who had at least some COVID-19 pandemic measurements in their country during the measurement period.

## Results

### Descriptive statistics

Descriptive statistics are reported in Table 1 for the whole sample and Table 2 for the accelerometer sample. Correlations between the main study variables are shown in Table 3. The average acceleration was 27.9 ± 19.9 milligravity varying from 23.8 in Sweden to 35.5 in Denmark. The intensity gradient was on average -2.4 ± 0.4 and the lowest intensity distribution was in Poland (− 2.53) and the highest in Sweden (− 2.28). Self-reported physical activity was on average 2.8 ± 1.0 (scale 1–4) with the lowest physical activity reported in Cyprus (2.2) and the highest in Finland (3.2). GDP varied from $116 000 in Luxemburg to $10 079 in Bulgaria. The number of policies for physical activity varied from 1 in Luxemburg to 20 in Sweden.

**Table 1:** Descriptive statistics for the whole sample

	N	Age (years)	Female (%)	Education	BMI	Chronic diseases	Self-reported PA	Quality of life	Personality traits					GDP	Policies
									N	E	O	A	C		
Austria	1566	72.3 ± 8.9	60.5	3.3 ± 1.3	26.5 ± 4.6	1.9 ± 1.7	2.9 ± 1.0	39.9 ± 5.4	2.5 ± 1.0	3.7 ± 1.0	3.6 ± 1.0	4.2 ± 0.8	3.6 ± 0.8	4.9	5
Belgium	2003	70.0 ± 9.7	55.7	3.2 ± 1.5	26.5 ± 4.6	2.0 ± 1.6	2.7 ± 1.0	38.7 ± 5.8	2.7 ± 1.0	3.5 ± 0.9	3.3 ± 1.0	4.1 ± 0.7	3.6 ± 0.8	4.5	7
Bulgaria	906	68.3 ± 9.4	60.6	2.8 ± 1.1	27.5 ± 4.7	1.8 ± 1.4	2.5 ± 1.1	33.6 ± 6.4	2.6 ± 0.9	3.8 ± 0.8	3.4 ± 0.6	4.4 ± 0.8	3.6 ± 0.9	1.0	6
Croatia	1187	68.3 ± 9.0	56.6	2.9 ± 1.3	27.7 ± 4.4	2.2 ± 1.7	2.9 ± 1.0	36.3 ± 6.0	2.8 ± 0.9	3.8 ± 0.8	2.9 ± 0.9	4.3 ± 0.7	3.6 ± 0.7	1.4	6
Cyprus	536	72.6 ± 10.0	61.2	2.2 ± 1.6	27.2 ± 4.6	2.3 ± 1.7	2.2 ± 1.1	34.8 ± 6.8	2.9 ± 1.2	3.0 ± 0.7	3.2 ± 0.8	4.5 ± 0.7	3.5 ± 1.1	2.8	6
Czech Rep	2714	71.5 ± 8.1	61.2	2.9 ± 1.2	28.2 ± 4.8	2.3 ± 1.7	2.7 ± 1.0	36.5 ± 5.5	2.8 ± 0.9	3.3 ± 0.8	3.4 ± 0.9	3.9 ± 0.8	3.5 ± 0.8	2.3	3
Denmark	2168	69.4 ± 9.3	54.2	3.7 ± 1.3	26.2 ± 4.5	1.7 ± 1.5	3.2 ± 0.9	41.2 ± 4.6	2.0 ± 1.0	4.0 ± 1.1	3.0 ± 1.1	4.3 ± 0.8	4.2 ± 0.8	6.1	10
Estonia	3024	71.7 ± 9.7	63.6	3.4 ± 1.2	28.1 ± 5.2	2.0 ± 1.7	2.7 ± 1.0	36.3 ± 6.3	2.5 ± 1.1	3.7 ± 1.1	3.7 ± 1.0	3.9 ± 0.9	3.6 ± 0.8	2.3	3
Finland	1160	68.4 ± 9.4	53.5	3.3 ± 1.6	27.1 ± 4.5	2.1 ± 1.6	3.2 ± 0.9	38.4 ± 5.2	2.3 ± 1.0	3.5 ± 1.0	3.7 ± 1.0	3.9 ± 0.9	4.1 ± 0.8	4.9	11
France	2476	71.0 ± 9.8	58.6	2.8 ± 1.7	26.4 ± 4.8	2.0 ± 1.6	2.7 ± 1.0	38.3 ± 5.9	2.9 ± 1.1	3.3 ± 0.8	3.3 ± 0.9	4.3 ± 0.7	3.7 ± 0.7	3.9	5
Germany	2878	69.7 ± 9.1	53.5	3.6 ± 1.1	27.0 ± 5.1	2.1 ± 1.7	3.0 ± 1.0	39.4 ± 5.4	2.6 ± 1.0	3.4 ± 1.0	3.5 ± 1.0	4.2 ± 0.8	3.5 ± 0.8	4.6	4
Greece	2998	69.5 ± 9.8	57.7	2.5 ± 1.6	27.3 ± 3.9	2.0 ± 1.6	2.7 ± 0.9	32.0 ± 5.6	3.6 ± 0.9	3.1 ± 0.7	3.0 ± 0.8	4.0 ± 0.7	3.5 ± 0.8	1.8	5
Hungary	772	70.2 ± 7.8	61.0	3.1 ± 1.0	27.8 ± 5.4	2.1 ± 1.6	2.3 ± 1.0	36.8 ± 6.7	2.6 ± 1.0	3.2 ± 0.6	3.2 ± 0.9	4.1 ± 0.7	3.3 ± 0.7	1.6	10
Israel	926	73.1 ± 8.8	59.7	3.0 ± 1.6	26.6 ± 4.8	2.0 ± 1.8	2.5 ± 1.1	35.9 ± 5.6	2.5 ± 0.9	3.5 ± 0.8	3.4 ± 0.9	4.1 ± 0.8	3.6 ± 0.9	4.4	-
Italy	2154	70.6 ± 9.7	56.4	2.0 ± 1.3	26.3 ± 4.1	1.7 ± 1.5	2.3 ± 1.1	34.8 ± 6.3	2.7 ± 0.9	3.2 ± 0.8	3.2 ± 0.9	4.0 ± 0.7	3.7 ± 0.7	3.2	4
Latvia	794	68.2 ± 10.1	63.1	3.5 ± 1.2	28.8 ± 5.3	1.8 ± 1.3	3.0 ± 1.0	33.9 ± 6.0	2.9 ± 1.0	3.1 ± 1.0	3.1 ± 0.5	4.0 ± 0.8	3.6 ± 0.8	1.8	2
Lithuania	1436	68.3 ± 10.5	62.9	3.5 ± 1.4	28.4 ± 5.3	2.1 ± 1.7	2.7 ± 1.1	33.9 ± 6.3	2.9 ± 1.0	3.2 ± 0.7	3.1 ± 1.0	3.9 ± 0.9	3.8 ± 0.8	2.0	10
Luxembourg	952	68.2 ± 8.8	54.7	2.8 ± 1.5	26.9 ± 4.8	2.3 ± 2.0	2.9 ± 1.0	40.4 ± 5.3	2.6 ± 1.1	3.6 ± 0.9	3.5 ± 1.0	4.3 ± 0.7	3.8 ± 0.8	11.6	1
Malta	805	68.5 ± 8.8	54.9	2.2 ± 1.4	30.2 ± 5.8	1.5 ± 1.3	2.3 ± 1.0	36.6 ± 5.2	2.7 ± 1.0	3.7 ± 1.0	3.0 ± 0.9	4.4 ± 0.7	3.5 ± 0.9	2.8	3
Netherlands	1935	70.8 ± 8.3	54.4	3.2 ± 1.4	26.3 ± 4.9	1.5 ± 1.4	3.2 ± 0.9	40.6 ± 4.9	-	-	-	-	-	5.2	4
Poland	2075	68.0 ± 9.5	56.0	2.8 ± 1.1	27.9 ± 4.8	2.5 ± 1.9	2.6 ± 1.0	36.6 ± 6.3	2.8 ± 0.8	3.3 ± 0.7	3.2 ± 0.8	3.9 ± 0.7	3.2 ± 0.7	1.6	3
Romania	1280	67.0 ± 9.4	57.5	2.5 ± 1.1	28.5 ± 5.1	1.6 ± 1.4	2.8 ± 1.1	-	2.9 ± 1.0	3.7 ± 0.9	3.2 ± 0.9	4.2 ± 0.8	3.6 ± 1.0	1.3	6
Slovakia	997	63.3 ± 8.3	55.4	3.0 ± 0.7	27.4 ± 4.3	1.1 ± 1.4	2.7 ± 0.9	36.0 ± 6.7	2.6 ± 0.9	3.4 ± 0.8	3.3 ± 0.6	4.1 ± 0.8	3.5 ± 0.8	1.9	2
Slovenia	2492	70.9 ± 9.2	58.8	3.0 ± 1.2	27.5 ± 4.5	1.9 ± 1.6	2.9 ± 1.0	38.8 ± 5.8	2.6 ± 0.9	3.6 ± 0.9	3.3 ± 0.6	4.2 ± 0.7	3.9 ± 0.7	2.6	4
Spain	2116	73.5 ± 9.9	56.9	1.7 ± 1.5	27.2 ± 4.6	2.2 ± 1.7	2.5 ± 1.0	37.5 ± 6.4	2.6 ± 1.1	3.5 ± 1.0	3.2 ± 1.0	4.1 ± 0.8	3.9 ± 0.8	2.7	3
Sweden	2353	73.2 ± 8.5	54.0	3.3 ± 1.5	26.0 ± 4.2	1.7 ± 1.5	3.1 ± 0.9	39.2 ± 5.0	2.2 ± 1.0	3.7 ± 1.0	3.1 ± 1.1	4.1 ± 0.9	4.1 ± 0.7	5.2	20
Switzerland	1903	71.5 ± 9.1	54.8	3.3 ± 1.2	25.5 ± 4.5	1.4 ± 1.4	3.1 ± 0.9	40.9 ± 4.7	2.5 ± 1.0	3.5 ± 0.9	3.6 ± 0.9	4.3 ± 0.7	3.7 ± 0.8	8.7	7
Total	46 617	70.3 ± 9.5	57.5	3.0 ± 1.4	27.2 ± 4.8	1.9 ± 1.6	2.8 ± 1.0	37.4 ± 6.3	2.7 ± 1.0	3.5 ± 0.9	3.3 ± 0.9	3.7 ± 0.8	4.1 ± 0.8	3.6	5.8

Means ± standard deviations presented unless otherwise noted. PA = Physical activity, N = Neuroticism, E = Extraversion, O = Openness, A = Agreeableness, C = Conscientiousness, GDP = Gross domestic product per capita / 10 000 ($), Policies = the number of national policies or action plans for the promotion of physical activity, Czech Rep. = Czech Republic

**Table 2:** Descriptive statistics for the accelerometer sample

	N	Age (years)	Female (%)	COVID (%)	Education	BMI	Chronic diseases	Average acceleration	Intensity gradient	Self-reported PA	Quality of life	Personality traits				
												N	E	O	A	C
Belgium	81	67.5 ± 9.9	58.0	6.2	3.3 ± 1.6	26.6 ± 5.1	2.0 ± 1.6	28.7 ± 20.8	-2.4 ± 0.4	2.8 ± 1.0	38.7 ± 6.6	2.9 ± 1.0	3.5 ± 0.8	3.2 ± 0.9	3.6 ± 0.8	4.1 ± 0.7
Czech Rep	105	69.7 ± 8.4	63.8	1.0	3.1 ± 1.2	28.1 ± 4.5	2.3 ± 1.7	21.9 ± 11.3	-2.4 ± 0.4	3.0 ± 0.9	37.0 ± 5.3	2.7 ± 0.9	3.3 ± 0.9	3.4 ± 1.0	3.6 ± 0.8	4.0 ± 0.8
Denmark	36	69.4 ± 9.2	47.2	11.1	3.5 ± 1.3	25.5 ± 3.8	1.4 ± 1.1	35.5 ± 23.6	-2.3 ± 0.4	3.1 ± 1.0	42.0 ± 3.6	2.0 ± 1.0	4.2 ± 0.9	2.6 ± 1.3	4.3 ± 0.7	4.1 ± 0.9
France	79	67.8 ± 9.7	68.4	10.1	3.2 ± 1.5	27.4 ± 4.6	1.9 ± 1.5	33.4 ± 23.3	-2.4 ± 0.4	2.7 ± 0.9	39.3 ± 5.4	3.0 ± 1.1	3.4 ± 0.8	3.5 ± 1.0	3.7 ± 0.7	4.4 ± 0.6
Germany	116	68.1 ± 9.6	56.0	30.2	3.8 ± 1.2	27.7 ± 5.1	2.0 ± 1.5	25.4 ± 13.3	-2.4 ± 0.3	3.1 ± 0.9	39.1 ± 5.6	2.8 ± 1.0	3.4 ± 1.0	3.5 ± 1.0	3.4 ± 0.9	4.1 ± 0.8
Italy	67	65.9 ± 9.0	56.7	25.4	2.1 ± 1.2	26.6 ± 4.9	1.3 ± 1.2	26.8 ± 13.9	-2.5 ± 0.4	2.7 ± 1.1	36.9 ± 5.6	2.6 ± 0.9	3.2 ± 0.7	3.6 ± 0.9	3.7 ± 0.8	4.1 ± 0.7
Poland	129	68.3 ± 8.2	56.6	0.0	2.7 ± 1.2	28.4 ± 5.5	2.6 ± 1.8	30.9 ± 27.1	-2.5 ± 0.5	2.6 ± 0.9	37.5 ± 5.4	2.9 ± 0.8	3.3 ± 0.6	3.1 ± 0.8	3.1 ± 0.6	4.0 ± 0.9
Slovenia	100	68.3 ± 8.6	64.0	1.0	3.1 ± 1.1	28.3 ± 5.1	1.9 ± 1.6	24.6 ± 13.0	-2.5 ± 0.4	3.0 ± 0.9	38.0 ± 5.9	2.6 ± 0.9	3.7 ± 0.9	3.3 ± 0.6	3.8 ± 0.7	4.1 ± 0.7
Spain	72	68.9 ± 9.3	55.6	1.4	2.3 ± 1.6	28.6 ± 4.6	1.6 ± 1.3	33.8 ± 28.0	-2.3 ± 0.6	2.7 ± 1.1	37.6 ± 5.4	2.4 ± 1.1	3.6 ± 1.1	3.3 ± 1.0	4.1 ± 0.8	4.2 ± 0.8
Sweden	71	72.8 ± 6.9	47.9	1.4	3.4 ± 1.4	26.1 ± 3.7	2.1 ± 1.6	23.8 ± 13.8	-2.3 ± 0.4	3.2 ± 0.7	39.7 ± 5.2	2.2 ± 0.9	3.7 ± 1.1	2.8 ± 1.0	4.1 ± 0.8	4.3 ± 0.8
Total	856	68.6 ± 9.0	58.3	8.5	3.0 ± 1.4	27.5 ± 4.9	2.0 ± 1.6	27.9 ± 19.9	-2.4 ± 0,4	2.9 ± 0.9	38.3 ± 5.6	2.7 ± 1.0	3.5 ± 0.9	3.3 ± 1.0	3.7 ± 0.8	4.1 ± 0.8

Means ± standard deviations presented unless otherwise noted. COVID = proportion of participants who wear accelerometer during a period in which a country-specific situation included recommendations or requirements not to leave house without exceptions, PA = Physical activity, N = Neuroticism, E = Extraversion, O = Openness, A = Agreeableness, C = Conscientiousness, Czech Rep. = Czech Republic

**Table 3:** Bivariate correlations between study variables

	Self-reported physical activity Whole sample	Self-reported physical activity Accelerometer sample	Average acceleration	Intensity gradient
Average acceleration		.08*		
Intensity gradient		.27***	.41***	
Neuroticism	−.12***	−.13***	−.02	−.07
Extraversion	.09***	.02	.04	.02
Openness	.10***	.06	.03	.01
Agreeableness	.06***	.10**	.01	.05
Conscientiousness	.14***	.16***	.06	.09**
Quality of life	.37***	.27***	.08*	.20***
GDP	.13***	.13***	.01	.13***
Number of policies	.11***	.11**	−.03	.11**
Age	−.31***	−.19***	−.09*	−.22***
Education	.22***	.18***	.01	.17***
Gender	−.07***	−.09*	−.03	−.14***

*p < .05, **p < .01, ***p < .001. Sample size accelerometer sample *n* = 838–856, whole sample *n* = 41,035 –46,617

### Association between individual-level factors, country-level factors, and physical activity outcomes

In the null model without predictors, the ICC indicated that 7.5% of the total variance in self-reported physical activity, 3.5% in average acceleration, and 1.9% in intensity gradient was attributable to between-country differences. The corresponding value for self-reported physical activity was 3.7% in the accelerometer sample. Based on the likelihood ratio tests, the effect of country was statistically significant for all outcomes (*x*^2^ = 2 731, df = 1, *p* < 0.05 for self-reported physical activity, *x*^2^ = 15.1, df = 1, *p* < 0.05 for average acceleration, and *x*^2^ = 7.3, df = 1, *p* < 0.05 for intensity gradient).

The random-intercept-random slope models were better than the random-intercept-only models when predicting self-reported physical activity in the whole sample (log-likelihood tests *p* < 0.01). This suggests that the effects of personality traits and quality of life on self-reported physical activity varied across countries. In the accelerometer sample, the random-intercept-only models were better for all outcomes suggesting similar associations between individual-level predictors and accelerometer-based physical activity in all ten countries. The random-intercepts-random-slope models did not achieve convergence for average acceleration with all predictors, and for intensity gradient with some predictors (neuroticism, openness, and agreeableness). The intercepts, variances of intercepts and slopes, covariances between intercepts and slopes, and -2 loglikelihoods for models are presented in supplementary materials (Tables S1 and S2).

Models predicting physical activity outcomes are shown in Table 4. All individual-level variables were associated with self-reported physical activity (Table 4, Model 1): Lower neuroticism and higher extraversion, openness, agreeableness, conscientiousness, and quality of life were associated with higher self-reported physical activity. Of country-level variables, higher GDP was associated with higher self-reported physical activity. The results remained the same after adjusting the models for health-related variables (Model 2). When all personality traits were estimated simultaneously (Model 3), lower neuroticism, higher extraversion, openness, and conscientiousness were associated with higher self-reported physical activity, but the association of agreeableness was not statistically significant. In a model including all variables simultaneously, higher openness, conscientiousness, and quality of life as well as the number of policies were associated with higher self-reported physical activity, but the associations of neuroticism and extraversion were not statistically significant (Model 4). The associations for self-reported physical activity were generally similar when the sample was limited to participants in the smaller accelerometer sample (Table 4): Lower neuroticism and higher agreeableness, conscientiousness, and quality of life were associated with higher frequency of self-reported physical activity (Model 1). In model including all variables simultaneously (Model 4), higher conscientiousness and quality of life were associated with higher self-reported physical activity.

**Table 4:** Parameter estimates from multilevel models predicting physical activity outcomes

	Average acceleration			Intensity gradient			Self-reported physical activity Whole sample			Self-reported physical activity Accelerometer sample		
	Estimate	S.E	*p*	Estimate	S.E	*p*	Estimate	S.E	*p*	Estimate	S.E	*p*
*Model 1*												
Neuroticism	−.404	.712	.571	−.012	.015	.421	−.089	.009	< .001	−.105	.032	.001
Extraversion	.951	.754	.208	.014	.016	.377	.067	.012	< .001	.016	.036	.652
Openness	.817	.735	.267	−.001	.015	.953	.063	.012	< .001	.056	.034	.103
Agreeableness	.271	.882	.759	.019	.018	.289	.053	.012	< .001	.105	.041	.010
Conscientiousness	1.352	.896	.132	.044	.019	.020	.181	.019	< .001	.191	.041	< .001
Quality of life	.233	.124	.062	.011	.003	< .001	.046	.001	< .001	.039	.005	< .001
GDP	.447	1.008	.667	.036	.015	.041	.059	.018	.004	.078	.044	.100
Number of policies	−.097	.271	.728	.011	.004	.023	.023	.012	.071	.027	.027	.031
*Model 2*												
Neuroticism	−.398	.724	.582	−.009	.015	.552	−.068	.009	< .001	−.085	.033	.010
Extraversion	1.152	.753	.126	.017	.016	.282	.064	.010	< .001	.027	.035	.450
Openness	.868	.736	.239	−.002	.015	.917	.060	.011	< .001	.052	.033	.118
Agreeableness	.387	.890	.664	.021	.018	.252	.044	.012	.001	.089	.040	.027
Conscientiousness	1.398	.898	.120	.042	.019	.026	.159	.018	< .001	.184	.040	< .001
Quality of life	.173	.131	.185	.009	.003	.001	.041	.001	< .001	.032	.006	< .001
GDP	.117	1.060	.914	.030	.017	.109	.054	.019	.007	.057	.046	.240
Number of policies	−.186	.280	.520	.009	.004	.064	.021	.013	.101	.023	.011	.068
*Model 3*												
Neuroticism	−.137	.724	.850	.0002	.016	.990	-.042	.008	< .001	−.051	.033	.122
Extraversion	.923	.778	.235	.012	.017	.472	.027	.008	.004	-.006	.036	.863
Openness	.773	.727	.288	−.003	.016	.836	.041	.009	< .001	.042	.033	.213
Agreeableness	.313	.895	.727	.019	.019	.314	.003	.011	.769	.056	.041	.173
Conscientiousness	1.228	.895	170	.037	.019	.056	.139	.017	< .001	.166	.041	< .001
*Model 4*												
Neuroticism	.069	.754	.927	.014	.016	.389	.003	.008	.748	−.027	.034	.424
Extraversion	729	.804	.364	−.002	.017	.885	−.001	.008	.859	-.045	.036	.215
Openness	.724	.746	.332	−.001	.016	.952	.027	.009	.011	.027	.034	.413
Agreeableness	.193	.921	.834	.012	.019	.545	−.0001	.009	.993	.039	.041	.351
Conscientiousness	.874	.918	.342	.024	.019	.207	.104	.013	< .001	.147	.041	< .001
Quality of life	.140	.138	.314	.008	.003	.005	.037	.001	< .001	.026	.006	< .001
GDP	.846	1.339	.541	.003	.024	.893	.001	.016	.930	−.003	.059	.955
Number of policies	−.362	.363	.341	.007	.006	.280	.025	.009	.012	.018	.016	.292

In model 1 and 2, every predictor was tested in a separate model. Models with individual-level predictors (neuroticism, extraversion, openness, agreeableness, conscientiousness, and quality of life) predicting self-reported physical activity in the whole sample are random-intercept-random-slope models. All other models (models with country-level predictors GDP and the number of policies, and all models in accelerometer sample) are random-intercept-only models. Models 1 adjusted for gender, education, and mean-centered age. Models 2 adjusted additionally for chronic diseases and body mass index. In model 3 and 4, all predictors and covariates (gender, education, mean-centered age, chronic diseases, and body mass index) were included simultaneously. *N* = 821–851 for accelerometer outcomes and *N* = 39,750–44,854 for self-reported physical activity

None of the individual- or country-level variables were associated with average acceleration. Higher conscientiousness and quality of life as well as higher GDP and the number of policies were associated with a flatter intensity gradient (i.e., with higher intensity distribution) (Model 1). After adjusting the models for health-related variables (Model 2), the associations between GDP and the number of policies and intensity gradient were not statistically significant; the association with conscientiousness and quality of life remained significant. When all personality traits were estimated simultaneously (Model 3), the association of conscientiousness was not statistically significant, and when all predictors were estimated simultaneously, only quality of life was associated with intensity gradient (Model 4). The supplementary analysis showed that the results were the same after the exclusion of participants who had at least some COVID-19 pandemic measurements in their country during the measurement period (Supplementary Table S3).

### Interactions between individual- and country-level factors

Because models for the accelerometer sample with 10 countries did not support the inclusion of the random slope in the models, the interaction models were estimated only for self-reported physical activity in the whole sample.

**Fig. 1:**
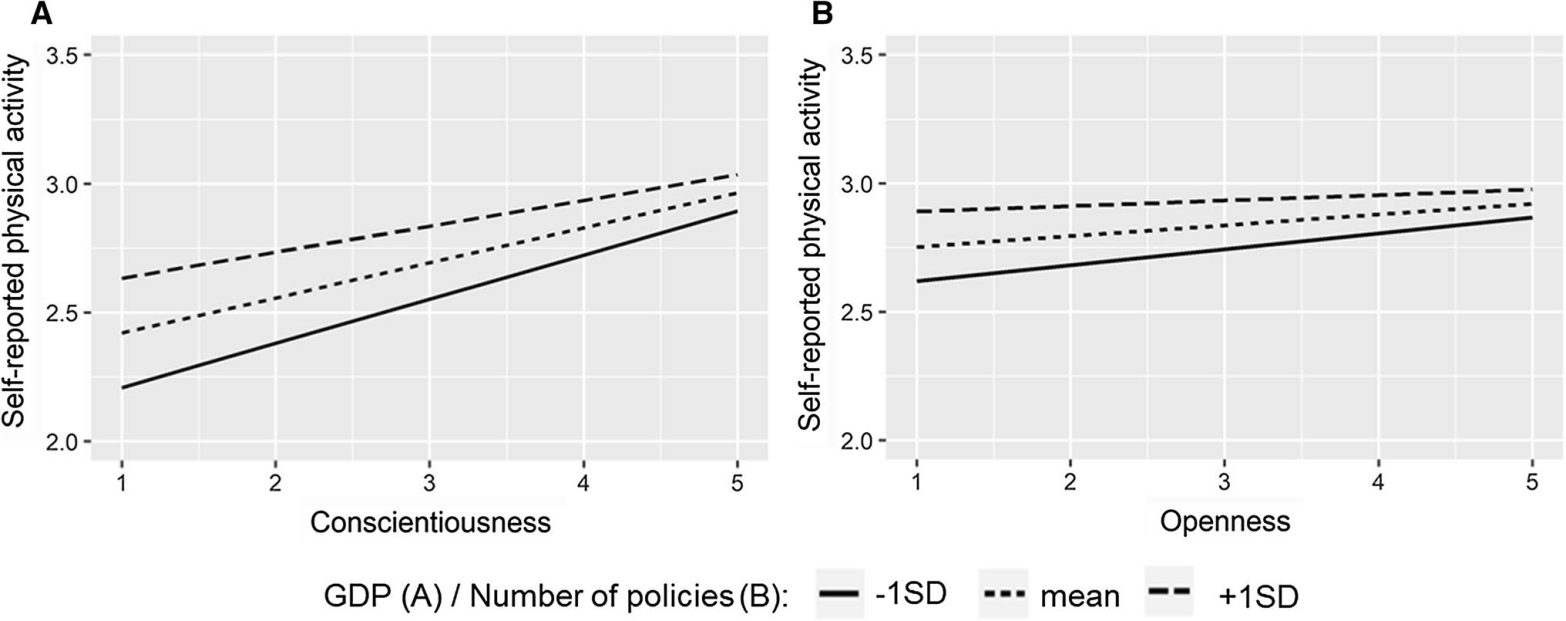
The moderator effect of GDP (**A**, **B**, and **C**) and the number of policies (**D**) on the association between personality traits and self-reported physical activity

Some cross-level interactions were found (Table 5): The association between neuroticism, extraversion, and conscientiousness and self-reported physical activity was stronger in countries with lower GDP (Model 1). After adjusting for health-related variables (Model 2), the interaction effect between neuroticism and GDP was not statistically significant anymore, and when all personality trait interaction were estimated simultaneously (Model 3), only the interaction of conscientiousness and GDP remained statistically significant (Fig. 1a). Openness had an interaction effect with the number of policies (Fig. 1b): The association between openness and self-reported physical activity was stronger in countries with fewer policies or action plans for the promotion of physical activity. This finding remained the same in all models.

**Table 5:** Parameter estimates from interaction multilevel models predicting self-reported physical activity (*N* = 39,750–42,664)

Cross-level interactions	Model 1			Model 2			Model 3		
	Estimate	S.E	*p*	Estimate	S.E	*p*	Estimate	S.E	*p*
Neuroticism*GDP	.008	.004	.029	.006	.003	.075	.003	.003	.301
Extraversion*GDP	−.012	.004	.016	−.010	.004	.015	−.006	.003	.069
Openness*GDP	−.002	.005	.697	−.001	.005	.879	.002	.004	.635
Agreeableness*GDP	−.005	.005	.349	−.004	.005	.476	.002	.005	.694
Conscientiousness*GDP	−.018	.007	.016	−.018	.007	.011	−.016	.006	.016
Quality of life*GDP	.000	.001	.901	.0004	.001	.488			
Neuroticism*Policies	.002	.002	.330	.002	.002	.448	.001	.002	.518
Extraversion*Policies	−.005	.003	.125	−.004	.002	.100	−.003	.002	.180
Openness*Policies	−.007	.003	.017	−.006	.002	.017	−.005	.002	.028
Agreeableness*Policies	.002	.003	.450	.002	.003	.563	.002	.003	.372
Conscientiousness*Policies	−.0002	.004	.945	−.001	.004	.826	.002	.004	.938
Quality of life*Policies	−.0002	.0003	.586	−.0001	.0003	.716			

In model 1 and 2, every interaction tested in a separate model. In model 3, interactions for personality traits tested simultaneously. All models are random-intercept-random-slope models. Models 1 adjusted for gender, education, and mean-centered age. Models 2 and 3 adjusted additionally for chronic diseases and body mass index

## Discussion

The purpose of this study was to test individual-level and country-level predictors of physical activity in a sample of older adults from European countries. The results showed that there were more associations between individual- and country-level factors and self-reported physical activity than with accelerometer-based physical activity. Furthermore, cross-level interactions suggested that associations between some personality traits and self-reported physical activity were stronger in countries with lower GDP.

All significant associations found in the present study were either with self-reported physical activity or intensity gradient; none of the individual- or country-level predictors were associated with average acceleration. While average acceleration captures all step-based activity across the measurement period and indicates volume of physical activity, it does not provide any information about intensity distribution. Individuals who spend a lot of time being sedentary but engage in a few vigorous exercise sessions may have the same average acceleration as individuals who spend most of their time in light physical activities (Rowlands et al. 2018). There is some evidence that while both overall activity level captured by average acceleration and intensity distribution have health effects, intensity distribution may be more important for functional capacity than average acceleration (Rowlands et al. 2018). The correlation (*r* = 0.27, *p* < 0.001) between intensity distribution and self-reported physical activity in the present study indicates that intensity distribution may be related more to participation in purposeful intensive physical activities, whereas average acceleration was weakly related to self-reported frequency of moderate and vigorous physical activities (*r* = 0.08, *p* = 0.018). Average acceleration may better reflect the amount of habitual light daily activities and individual- and country-level factors assessed in the present study were not related to average acceleration. Further studies focusing on environmental level predictors may shed light on determinants beyond overall physical activity volume.

The results were consistent with previous studies on personality traits and self-reported physical activity (Wilson and Dishman 2015; Sutin et al. 2016). These results were relatively consistent in both the full sample from 27 countries and the subsample from 10 countries indicating that the same pattern could be found in a subsample with lower power. In addition, the results were consistent with previous findings that personality traits have stronger associations with self-reported than accelerometer-based physical activity (Wilson et al. 2015; Kekäläinen et al. 2020a, b): Of the five personality traits, only conscientiousness was positively associated with intensity distribution. Even though the associations between personality traits and self-reported physical activity may be partly explained by common-method variance related to self-reported questionnaires (Podsakoff et al. 2003), personality traits are also consistently associated with objective physical performance or functioning indicators that do not share method variance, such as walking speed, VO2 max, and grip strength (Terracciano et al. 2013; Stephan et al. 2018, 2022; Kekäläinen et al. 2020b). Thus, these findings may be related more to different aspects of physical activity captured by questionnaires and devices. The predictive value of personality traits on physical activity may go through different sociocognitive characteristics, such as exercise preferences, intentions, motivations, and attitudes, that are rather related to purposeful exercise (captured by self-reported activity) than all daily activity (captured by accelerometers) (Wilson and Rhodes 2021).

The positive association between quality of life and both self-reported physical activity and accelerometer-based intensity distribution is consistent with previous studies, even though these studies have focused on health-related quality of life (Anokye et al. 2012; Sansano-Nadal et al. 2021). The CASP-questionnaire used in the present study differs from typical health-related quality of life questionnaires by focusing on the satisfaction of basic psychological needs (control, autonomy, self-realization, and pleasure) instead of components of health-related functioning (Hyde et al. 2003). The association between quality of life and physical activity is likely bidirectional: physical activity can improve quality of life through multiple pathways including improvements in physical functioning and depressive symptoms (Raafs et al. 2020), but the fulfilment of basic needs, such as control and autonomy, is also a prerequisite for participation in physical activity (Hagger and Chatzisarantis 2008). In addition, the experiences of control and autonomy may also mediate the association between personality traits and physical activity (Wilson and Rhodes 2021). This pathway may explain why quality of life was more consistently associated with physical activity in models including personality traits and quality of life simultaneously, especially because quality of life should be a more proximal predictor of physical activity, whereas personality should be more distal. In addition, it is important to note that quality of life was measured with 12 items, whereas each trait was measured with only two items, which likely reduced the predictive power or personality, particularly compared to the more comprehensive 12-item measure.

In line with ecological models (Sallis et al. 2006) and previous studies (Van Tuyckom 2011; Cameron et al. 2013), GDP and the number of policies and action plans for physical activity were associated with physical activity. These associations were relatively consistent with self-reported physical activity and intensity distribution. The lack of association between GDP and average acceleration in the present study is also consistent with previous findings that suggest that GDP is associated only with self-reported leisure time physical activity and not total physical activity (Cameron et al. 2013). As discussed above, both self-reported questionnaire and intensity distribution in the present study may capture more purposeful exercise activities, whereas average acceleration is an indicator of total physical activity. Higher GDP may be related to better infrastructure and sports facilities that provide opportunities for leisure physical activities (Schüz et al. 2012), while the number of policies and action plans for physical activity may indicate how well a national culture supports physical activity. However, as countries are at different points in their efforts to increase physical activity (World Health Organization 2018), in some countries the number of policies may also indicate a higher need for policies. Moreover, the associations of GDP and policies with physical activity attenuated when individual-level differences in personality traits and quality of life were taken into account. This suggests that these country-level factors may be less relevant for individuals´ physical activity levels than their personal resources captured by quality of life and personality traits.

No differences between countries in predictors of accelerometer-based physical activity were found in the small sample of ten countries. Nonetheless, some country-level moderator effects were found in the whole sample for self-reported physical activity. The association between conscientiousness and physical activity was stronger in countries with lower GDP. Similar interactions were found for extraversion and neuroticism, but these attenuated when other personality traits were taken into account. These results are consistent with previous findings that found stronger associations between personality traits and cognitive functioning in European countries with lower GDP (Luchetti et al. 2021) and also with findings that suggest a stronger role of socio-cognitive factors on physical activity when environmental facilities are poorer (Carlson et al. 2012; Ding et al. 2012). It seems that differences between individuals are more important in less advantageous environments. With fewer resources that support physical activity of the whole community, individual differences in personality seem to play a stronger role in achieving a higher level of physical activity. A single interaction effect with the number of policies was found suggesting a stronger association between openness and physical activity in countries with fewer policies and action plans for physical activity. Even though this single interaction may be due to chance, it is in line with found interactions between other personality traits and GDP suggesting that openness may be a resource for physical activity participation in an environment that is less supportive for physical activity.

The main strength of this study is the large sample of over 40,000 middle-aged and older adults from 27 European countries, and the inclusions of both self-report and accelerometer-based measures of physical activity. All variables used in the present study were based on harmonized questionnaires or accelerometer data that offer the possibility for comparisons between countries. Even though the accelerometer data were available only from a small subsample, this is one of the first studies to investigate associations between country-level factors and accelerometer-based physical activity. We used relatively new accelerometer-based variables, i.e., average acceleration and intensity distribution. Together, these variables provide a good description of different aspects of daily physical activity and are standardized measures suitable for comparison across studies (Rowlands et al. 2018).

It is unfortunate that due to the COVID-19 pandemic, the data collection was terminated and both the whole sample and the accelerometer subsample were smaller than originally targeted. The small sample size and smaller variation between 10 countries compared to the whole sample of 27 countries are likely to explain the lack of between-country differences in accelerometer-based physical activity. This study was also limited to cross-sectional analyses, and thus it is not possible to draw conclusions about longitudinal associations or causal relationships. In addition, there were some limitations in the questionnaires. Both personality traits (BFI-10) and quality of life (CASP-12) were assessed with shortened versions of original questionnaires that may produce smaller effect sizes than longer questionnaires. Self-reported physical activity was based on only two questions on the frequency of participation in moderate or vigorous physical activities. Country-level indicators also have their limitations. GDP is a general indicator of a country´s economic situation and for example, does not reveal regional differences within a country. The number of policies and action plans for physical activity indicates only the existence of these policies and not their implementation. In addition, some policies may not be relevant for older adults (e.g., policies and action plans targeting children or younger adults). Future studies including also low-income countries are needed to strengthen the understanding of country-level differences.

Despite these limitations, this study makes an important contribution to the research field by investigating how relatively stable and persistent individual-level factors and country-level factors are associated with physical activity. The results suggest that both individual- and country-level factors are related to participation in more intensive physical activities and the role of personality traits seems to be stronger in countries with relatively poorer economic conditions. This implies that persons with less resilient personality traits in terms of physical activity living in less advantageous countries are at the highest risk for physical inactivity and could be targeted in interventions.

## Supplementary Information

Below is the link to the electronic supplementary material.

Supplementary file1 (DOCX 39 KB)

## Data Availability

The SHARE data are available in a public, open access repository for registered users (http://www.share-project.org/home0.html).
