# Supplementary material for: Individual and country-level factors associated with self-reported and accelerometer-based physical activity in old age: a cross-national analysis of European countries
Source: Eur J Ageing. 2022 Oct 21;19(4):1529–42. doi: 10.1007/s10433-022-00737-8 (PMC9589794; doi:10.1007/s10433-022-00737-8)
Supplement: Supplementary file 1 — Supplementary file1 (DOCX 39 KB) [file 10433_2022_737_MOESM1_ESM.docx]

**Individual and country-level factors associated with self-reported and accelerometer-based physical activity in old age: A cross-national analysis of European countries**

**Supplementary materials**

**Table S1**. Parameter estimates and -2 -likelihood values from multilevel models predicting accelerometer-based physical activity (N = 821–851).

|  | Average acceleration | | | Intensity gradient | | | | | |
| --- | --- | --- | --- | --- | --- | --- | --- | --- | --- |
|  | Random-intercept  model | | | Random-intercept  model | | | Random-intercept-random-slope model | | |
|  | Estimate | S.E. | p | Estimate | S.E. | p | Estimate | S.E. | p |
| **Neuroticism** | -.404 | .712 | .571 | -.012 | .015 | .421 |  |  |  |
| Intercept | 28.167 | 2.980 | <.001 | -2.543 | .062 | <.001 |  |  |  |
| Intercept variance | 12.895 | 8.154 | .114 | .004 | .003 | .146 |  |  |  |
| **Extraversion** | .951 | .754 | .208 | .014 | .016 | .377 | .015 | .020 | .494 |
| Intercept | 23.056 | 3.381 | <.001 | -2.632 | .072 | <.001 | -2.636 | .081 | <.001 |
| Intercept variance | 12.298 | 7.762 | .113 | .004 | .003 | .146 | .018 | .026 | .503 |
| Slope variance |  |  |  |  |  |  | .001 | .002 | .472 |
| I & S covariance |  |  |  |  |  |  | -.004 | .007 | .530 |
| -2 likelihoood |  |  |  | 910 |  |  | 908 |  |  |
| **Openness** | .817 | .735 | .267 | -.001 | .015 | .953 |  |  |  |
| Intercept | 24.497 | 3.070 | <.001 | -2.576 | .064 | <.001 |  |  |  |
| Intercept variance | 13.469 | 8.466 | .112 | .004 | .003 | .137 |  |  |  |
| **Agreeableness** | .271 | .882 | .759 | .019 | .018 | .289 |  |  |  |
| Intercept | 26.199 | 3.901 | <.001 | -2.654 | .080 | <.001 |  |  |  |
| Intercept variance | 12.945 | 8.170 | .113 | .004 | .003 | .174 |  |  |  |
| **Conscientiousness** | 1.352 | .896 | .132 | .044 | .019 | .020 | .046 | .021 | .061 |
| Intercept | 21.556 | 4.274 | <.001 | -2.765 | .089 | <.001 | -2.777 | .090 | <.001 |
| Intercept variance | 12.905 | 8.177 | .114 | .004 | .003 | .158 | .007 | .030 | .811 |
| Slope variance |  |  |  |  |  |  | .001 | .002 | .628 |
| I & S covariance |  |  |  |  |  |  | -.002 | .008 | .753 |
| -2 likelihoood |  |  |  | 907 |  |  | 905 |  |  |
| **Quality of life** | .233 | .124 | .062 | .011 | .003 | <.001 | .011 | .003 | .004 |
| Intercept | 18.562 | 4.934 | <.001 | -2.965 | .101 | <.001 | -2.971 | .109 | <.001 |
| Intercept variance | 12.225 | 7.859 | .120 | .004 | .003 | .145 | .020 | .048 | .680 |
| Slope variance |  |  |  |  |  |  | .00001 | .00003 | .689 |
| I & S covariance |  |  |  |  |  |  | -.0005 | .001 | .711 |
| -2 likelihoood |  |  |  | 864 |  |  | 864 |  |  |
| **GDP** | .447 | 1.008 | .667 | .036 | .015 | .041 |  |  |  |
| Intercept | 25.400 | 4.001 | <.001 | -2.697 | .062 | <.001 |  |  |  |
| Intercept variance | 13.096 | 8.161 | .109 | .002 | .002 | .300 |  |  |  |
| **The number of policies** | -.097 | .271 | .728 | .011 | .004 | .023 |  |  |  |
| Intercept | 27.447 | 2.587 | <.001 | -2.637 | .044 | <.001 |  |  |  |
| Intercept variance | 12.963 | 8.173 | .113 | .002 | .002 | .293 |  |  |  |

Models adjusted for gender, education, and mean-centered age. Random-intercept-random-slope models are not presented if the model convergence was not achieved. I & S covariance = Intercept and slope covariance.

**Table S2.** Parameter estimates and -2 -likelihood values from multilevel models predicting self-reported physical activity

|  | Whole sample | | | | | | | Accelerometer sample | | |
| --- | --- | --- | --- | --- | --- | --- | --- | --- | --- | --- |
|  | Random-intercept  model | | | Random-intercept-  random-slope model | | | Random-intercept  model | | | |
|  | Estimate | S.E. | p | Estimate | S.E. | p | Estimate | | S.E. | p |
| **Neuroticism** | -.081 | .005 | <.001 | -.089 | .009 | <.001 | -.105 | | .032 | .001 |
| Intercept | 2.697 | .052 | <.001 | 2.721 | .045 | <.001 | 2.897 | | .137 | <.001 |
| Intercept variance | .060 | .017 | <.001 | .043 | .014 | .002 | .027 | | .017 | .107 |
| Slope variance |  |  |  | .002 | .001 | .012 |  | |  |  |
| I & S covariance |  |  |  | .002 | .002 | .438 |  | |  |  |
| -2 likelihoood | 109 696 |  |  | 109 657 |  |  |  | |  |  |
| **Extraversion** | .058 | .005 | <.001 | .067 | .012 | <.001 | .016 | | .036 | .652 |
| Intercept | 2.264 | .053 | <.001 | 2.231 | .074 | <.001 | 2.528 | | .163 | <.001 |
| Intercept variance | .062 | .017 | <.001 | 0.130 | .040 | .001 | .033 | | .019 | .094 |
| Slope variance |  |  |  | .003 | .001 | .009 |  | |  |  |
| I & S covariance |  |  |  | -.015 | .006 | .013 |  | |  |  |
| -2 likelihoood | 109 714 |  |  | 109 668 |  |  |  | |  |  |
| **Openness** | .064 | .005 | <.001 | .063 | .012 | <.001 | .056 | | .034 | .103 |
| Intercept | 2.279 | .053 | <.001 | 2.282 | .067 | <.001 | 2.427 | | .144 | <.001 |
| Intercept variance | .064 | .018 | <.001 | .103 | .031 | .001 | .035 | | .021 | .091 |
| Slope variance |  |  |  | .003 | .001 | .007 |  | |  |  |
| I & S covariance |  |  |  | -.011 | .005 | .024 |  | |  |  |
| -2 likelihoood | 109 530 |  |  | 109 465 |  |  |  | |  |  |
| **Agreeableness** | .052 | .006 | <.001 | .053 | .012 | <.001 | .105 | | .041 | .010 |
| Intercept | 2.274 | .054 | <.001 | 2.268 | .072 | <.001 | 2.191 | | .180 | <.001 |
| Intercept variance | .061 | .017 | <.001 | .117 | .036 | .001 | .027 | | .017 | .112 |
| Slope variance |  |  |  | .003 | .001 | .008 |  | |  |  |
| I & S covariance |  |  |  | -.013 | .006 | .021 |  | |  |  |
| -2 likelihoood | 109 819 |  |  | 109 776 |  |  |  | |  |  |
| **Conscientiousness** | .173 | .006 | <.001 | .181 | .019 | <.001 | .191 | | .041 | <.001 |
| Intercept | 1.744 | .058 | <.001 | 1.711 | .109 | <.001 | 1.784 | | .196 | <.001 |
| Intercept variance | .068 | .019 | <.001 | .287 | .087 | .001 | .030 | | .018 | .099 |
| Slope variance |  |  |  | .009 | .003 | .002 |  | |  |  |
| I & S covariance |  |  |  | -.043 | .014 | .003 |  | |  |  |
| -2 likelihoood | 109 031 |  |  | 108 903 |  |  |  | |  |  |
| **Quality of life** | .046 | .001 | <.001 | .046 | .001 | <.001 | .039 | | .005 | <.001 |
| Intercept | .891 | .052 | <.001 | .907 | .061 | <.001 | 1.162 | | .223 | <.001 |
| Intercept variance | .050 | .014 | <.001 | .073 | .028 | .009 | .030 | | .018 | .090 |
| Slope variance |  |  |  | .00003 | .00001 | .020 |  | |  |  |
| I & S covariance |  |  |  | -.0009 | .0005 | .094 |  | |  |  |
| -2 likelihoood | 109 989 |  |  | 109 964 |  |  |  | |  |  |
| **GDP** | .059 | .018 | .004 |  |  |  | .078 | | .044 | .100 |
| Intercept | 2.250 | .080 | <.001 |  |  |  | 2.304 | | .173 | <.001 |
| Intercept variance | .051 | .014 | <.001 |  |  |  | .023 | | .015 | .122 |
| **The number of policies** | .023 | .012 | .071 |  |  |  | .027 | | .027 | .031 |
| Intercept | 2.320 | .088 | <.001 |  |  |  | 2.416 | | .109 | <.001 |
| Intercept variance | .060 | .017 | <.001 |  |  |  | .018 | | .012 | .133 |

Models adjusted for gender, education, and mean-centered age. The whole sample N = 39,750–44,854, the accelerometer sample N = 821–851. Random-intercept-random-slope models are not presented for the accelerometer sample because the model convergence was not achieved. I & S covariance = Intercept and slope covariance.

**Table S3.** Parameter estimates from multilevel models predicting accelerometer-based physical activity in a sample without COVID-19 pandemic measurements in their country during the measurement-period (n = 770–778).

|  | Average acceleration | | | Intensity gradient | | |
| --- | --- | --- | --- | --- | --- | --- |
|  |  | | |  | | |
|  | Estimate | S.E. | p | Estimate | S.E. | p |
| **Model 1** |  |  |  |  |  |  |
| Neuroticism | -.443 | .754 | .557 | -.012 | .016 | .468 |
| Extraversion | 1.060 | .793 | .181 | .018 | .017 | .297 |
| Openness | .788 | .789 | .318 | -.003 | .017 | .976 |
| Agreeableness | .145 | .944 | .878 | .022 | .020 | .261 |
| Conscientiousness | 1.779 | .960 | .064 | .045 | .020 | .025 |
| Quality of life | .220 | .132 | .095 | .010 | .003 | <.001 |
| GDP | .505 | .985 | .619 | .040 | .016 | .029 |
| Number of policies | -.093 | .264 | .732 | .011 | .004 | .024 |
| **Model 2** |  |  |  |  |  |  |
| Neuroticism | -.443 | .766 | .563 | -.010 | .016 | .546 |
| Extraversion | 1.264 | .793 | .111 | .021 | .017 | .213 |
| Openness | .818 | .791 | .301 | -.004 | .017 | .812 |
| Agreeableness | .252 | .956 | .792 | .024 | .020 | .224 |
| Conscientiousness | 1.779 | .961 | .064 | .044 | .020 | .030 |
| Quality of life | .148 | .138 | .285 | .009 | .003 | .002 |
| GDP | .165 | 1.048 | .878 | .034 | .018 | .080 |
| Number of policies | -.186 | .274 | .512 | .010 | .005 | .062 |
| **Model 3** |  |  |  |  |  |  |
| Neuroticism | -.138 | .770 | .858 | .001 | .017 | .944 |
| Extraversion | 1.060 | .817 | .195 | .017 | .017 | .322 |
| Openness | .664 | .782 | .396 | -.007 | .017 | .672 |
| Agreeableness | .165 | .961 | .863 | .023 | .021 | .268 |
| Conscientiousness | 1.635 | .955 | .087 | .039 | .021 | .060 |
| **Model 4** |  |  |  |  |  |  |
| Neuroticism | .039 | .805 | .962 | .016 | .017 | .341 |
| Extraversion | .890 | .843 | .291 | .003 | .017 | .874 |
| Openness | .620 | .803 | .440 | -.004 | .017 | .830 |
| Agreeableness | .097 | .987 | .922 | .016 | .021 | .437 |
| Conscientiousness | 1.309 | .981 | .182 | .026 | .021 | .209 |
| Quality of life | .109 | .146 | .455 | .008 | .003 | .010 |
| GDP | .943 | 1.304 | .485 | .009 | .025 | .729 |
| Number of policies | -.390 | .351 | .292 | .007 | .007 | .350 |

In model 1 and 2, every predictor was tested in a separate model. Models 1 adjusted for gender, education, and mean-centered age. Models 2 adjusted additionally for chronic diseases and body mass index. Models 1 adjusted for gender, education, and mean-centered age. Models 2 adjusted additionally for chronic diseases and body mass index. In model 3 and 4, all predictors and covariates (gender, education, mean-centered age, chronic diseases, and body mass index) were included simultaneously.
